# Development of Sustainable and Active Food Packaging Materials Composed by Chitosan, Polyvinyl Alcohol and Quercetin Functionalized Layered Clay

**DOI:** 10.3390/polym16060727

**Published:** 2024-03-07

**Authors:** Chengyu Wang, Long Mao, Bowen Zheng, Yujie Liu, Jin Yao, Heping Zhu

**Affiliations:** 1 Key Laboratory of Advanced Packaging Materials and Technology of Hunan Province, Hunan University of Technology, Zhuzhou 412007, China; d21080500002@hut.edu.cn (C.W.); yaojin@hut.edu.cn (J.Y.); 2 Fujian Provincial Key Laboratory of Functional Materials and Applications, Xiamen University of Technology, Xiamen 361024, China; 13959280885@163.com (B.Z.); lyj1055348378@163.com (Y.L.)

**Keywords:** active packaging, chitosan, quercetin, layered clay, polyvinyl alcohol

## Abstract

In order to solve the problems of insufficient active functions (antibacterial and antioxidant activities) and the poor degradability of traditional plastic packaging materials, biodegradable chitosan (CS)/polyvinyl alcohol (PVA) nanocomposite active films reinforced with natural plant polyphenol-quercetin functionalized layered clay nanosheets (QUE-LDHs) were prepared by a solution casting method. In this study, QUE-LDHs realizes a combination of the active functions of QUE and the enhancement effect of LDHs nanosheets through the deposition and complexation of QUE and copper ions on the LDHs. Infrared and thermal analysis results revealed that there was a strong interface interaction between QUE-LDHs and CS/PVA matrix, resulting in the limited movement of PVA molecules and the increase in glass transition temperature and melting temperature. With the addition of QUE-LDHs, the active films showed excellent UV barrier, antibacterial, antioxidant properties and tensile strength, and still had certain transparency in the range of visible light. As QUE-LDHs content was 3 wt%, the active films exhibited a maximum tensile strength of 58.9 MPa, representing a significant increase of 40.9% compared with CS/PVA matrix. Notably, the UV barrier (280 nm), antibacterial (*E. coli*) and antioxidant activities (DPPH method) of the active films achieved 100.0%, 95.5% and 58.9%, respectively. Therefore, CS/PVA matrix reinforced with QUE-LDHs has good potential to act as an environmentally and friendly active packaging film or coating.

## 1. Introduction

In order to maintain food quality and protect food from extraneous contamination, food packaging plays a crucial role, as it ensures the food industry provides safe edible products [1]. Packaging provides more than just basic physical protection and barrier properties [2,3]; however, the packaging materials commonly applied in the food industry are made from non-degradable polymers that lack active functions (such as antibacterial and antioxidant activities), leading to environmental pollution or even food spoilage [4]. With the increasingly serious environmental problems, researchers have begun to pay attention to biodegradable polymers that can extend the shelf life of food. Therefore, various environmentally friendly active packaging materials have emerged and attracted widespread attention [3].

As the only cationic polysaccharide in biomass resources, chitosan (CS) ranks as the second-largest natural polymer in nature after cellulose [5]. CS exhibits excellent film-forming abilities, broad-spectrum antibacterial activity, and easy availability [6]. However, CS also demonstrates a moisture absorption ability and brittleness [7]. Blending CS with other synthetic polymer materials can effectively improve its shortcomings [8,9,10]. Polyvinyl alcohol (PVA) has found widespread use in packaging, biomedicine, and other fields due to its good film-forming properties, water solubility, mechanical strength, transparency, and degradability [7,9]. The development of biodegradable active food packaging materials with excellent performance through the blending of PVA and CS has been extensively studied [11,12,13], and this approach takes advantage of the broad spectrum of antibacterial activity and availability of CS, as well as the excellent film forming properties and stability of PVA [7,14].

Combined with literature reports [9,13], to further enhance the comprehensive performance of CS/PVA matrix, it is generally necessary to modify them by introducing a third-phase with active functions. Natural plant polyphenols, such as tannins, anthocyanin, quercetin, tea polyphenols, etc., exhibit excellent biological activity as phenolic secondary metabolites in plants [15,16]. These plant polyphenols demonstrate active functions, including antibacterial, antioxidant, and UV barrier properties, and have been applied to the modification of CS/PVA matrix [7,9]. Koosha et al. [9] studied CS/PVA composite active films doped with black carrot anthocyanin and layered clay (bentonite). They explored the application potential of anthocyanin as a natural pH indicator and bentonite as a nanofiller in the field of active packaging. The results showed that the addition of bentonite alone significantly reduces the mechanical properties of CS/PVA composite films. Although the mechanical and antibacterial properties of CS/PVA composite films were improved by the further addition of anthocyanins, the gas barrier properties of CS/PVA composite films were significantly affected. Haghighi et al. [13] prepared CS/PVA composite films enriched with ethyl lauroyl arginate (LAE) for food packaging applications. Although the addition of LAE improved the antibacterial activity and UV barrier properties of CS/PVA composite films, the mechanical and water vapor barrier properties were deteriorated to a certain extent. Therefore, to improve the comprehensive performance of the CS/PVA matrix, it is important to realize the multi-functionality of the third phase.

Inspired by the super-adhesion and versatility of marine adhesion proteins, the functionalization of material surfaces (films, nanoparticles, etc.) can be achieved by using catechol compounds similar in structure to adhesion proteins to form functional coatings (such as polydopamine (PDA) and tannin-metalion (TA-metal) coordination compounds) [17,18]. In our previous work [19,20], a simple and green method has been reported to prepare natural plant polyphenols containing active catechol functionalized layered clay nanosheets (Layered double hydroxides, LDHs), such as LDHs@TA-Fe^3+^ and LDHs@anthocyanin-Cu^2+^. This biomimetic modification method, based on mussels, can not only increase the interfacial compatibility between the LDHs and polymer matrix, thereby better utilizing the natural barrier properties and enhancing effects of LDHs nanosheets, but also achieve effective loading of natural active substances, endowing LDHs with antibacterial and antioxidant activities [20]. Quercetin (QUE) in natural plant polyphenols contains rich catechol groups, and it is currently yields the highest content of dietary flavonoids and polyphenols in fruits and vegetables, with good biological activity and medicinal value [21]. Therefore, the surface functionalization of LDHs using QUE can effectively combine the functions of LDHs and QUE, improving the versatility of the third phase (antibacterial, antioxidant activity, enhancement, etc.).

Based on the above analysis, this study attempted to use QUE to functionalize the LDHs to realize a combination of the active functions of QUE and the enhancement effect of LDHs nanosheets. QUE functionalized LDHs (QUE-LDHs) were synthesized by adsorption and complexation of QUE and Cu^2+^ on the surface of LDHs. Further, CS/PVA matriices reinforced with QUE-LDHs were prepared for the first time by the solution casting method. The influence of QUE-LDHs on the chemical structure, thermal, crystallization, mechanical, optical, antibacterial and antioxidant properties of CS/PVA matriices was evaluated to determine their feasibility for food active packaging.

## 2. Materials and Methods

### 2.1. Materials and Chemicals

QUE (purity ≥ 97%), copper chloride dihydrate (CuCl_2_·2H_2_O, purity ≥ 99.99%), CS (M_W_ = 30,000 Da, 85% deacetylation), 2,2-diphenyl-1-picrylhydrazyl (DPPH, purity ≥ 96%), ethanol (AR), methanol (AR) were provided by Shanghai Makclin Biochemical Technology Co., Ltd. (Shanghai, China). Ethylene-modified PVA (EXCEVAL™ AQ-4104, alcoholization degree 98–99 mol%) was purchased by Kuraray Co., Ltd. (Osaka, Japan). 2,2′-azinobis(3-ethylbenzothiazoline-6-sulfonic acid ammonium salt) (ABTS, purity = 98%), potassium persulfate (purity = 99.99%) were provided by Shanghai Aladdin Biochemical Technology Co., Ltd. (Shanghai, China). Phosphate buffer solution (PBS, 0.1 M, pH = 7.4) was purchased from Phygene Biotechnology Co., Ltd. (Fuzhou, China). LDHs was prepared by the hydrothermal reaction of metal salts with urea (particle size: ~1500 nm, thickness: ~40 nm) [20]. All chemical reagents are used directly without undergoing secondary treatment or purification.

### 2.2. Synthesis of QUE-Functionalized LDHs (QUE-LDHs)

LDHs (50 mg) was evenly dispersed in ethanol (100 mL) by ultrasonic treatment. Subsequently, QUE (50 mg) was added to the LDHs dispersion at room temperature and the dissolution of QUE was accelerated by mechanical stirring. Next, CuCl_2_·2H_2_O (28 mg) was dissolved in deionized water (50 mL). The prepared CuCl_2_ aqueous solution was then slowly poured into the LDHs dispersion, and the reaction was carried out at room temperature for 4 h. After the reaction, the reaction liquid was centrifuged with ethanol once and with deionized water twice. Finally, dark brown QUE-LDHs was obtained by a freeze-drying method. The characterization methods of QUE-LDHs have been given in the Supporting Information.

### 2.3. Preparation of QUE-LDHs/CS/PVA Nanocomposite Active Films

To enhance the water solubility and interfacial binding of CS, this study employed active catechol groups to further functionalize CS by carbodiimide coupling [22]. The composition and abbreviation of QUE-LDHs/CS/PVA nanocomposite active films are presented in Table 1. A certain amount of QUE-LDHs was dispersed in the deionized water (14 mL) and sonicated to disperse evenly. Then, CS (70 mg) was dissolved in the QUE-LDHs dispersion and the dissolution of CS was accelerated by mechanical stirring. Subsequently, PVA powder (630 mg) was added to the QUE-LDHs dispersion and heated to 95 °C under magnetic stirring until PVA powder was completely dissolved to obtain the active film-forming solution. The active film-forming solution was then sonicated to remove air bubbles. Finally, the above active film-forming solution was poured into a horizontally placed PTFE mold and transferred to an oven at 45 °C for drying for 24 h to obtain the homogeneous QUE-LDHs/CS/PVA nanocomposite active films. Before the subsequent tests, all the films were stored at 24 °C and 50% humidity for 48 h. For further information regarding the characterization of QUE-LDH/CS/PVA nanocomposite active films, please refer to the Supporting Information.

## 3. Results and Discussion

### 3.1. Chemical Structure of QUE-LDHs

**Figure 1:**
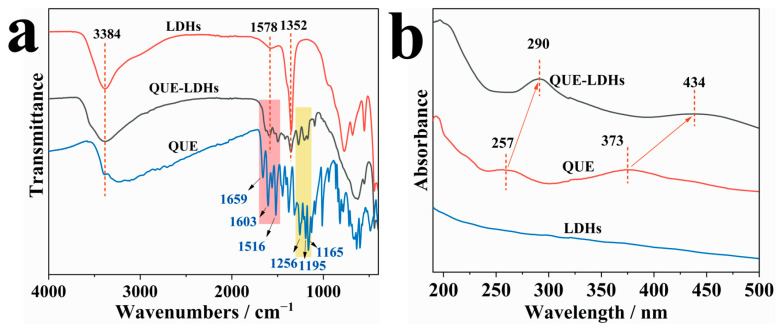
(**a**) FT-IR spectra and (**b**) UV-Vis spectra of QUE-LDHs, QUE and LDHs.

Figure 1 shows the FT-IR and UV-Vis spectra of QUE-LDHs, QUE and LDHs. In Figure 1a, the absorption peaks at 3384, 1578, and 1352 cm^−1^ in LDHs are due to the stretching and bending vibration of O-H bonds and the stretching vibration of interlayer carbonate ions, respectively [20]. Compared with LDHs, new absorption peaks of C=C groups (marked in red) and C-O groups (marked in yellow) in the QUE-LDHs are attributed to the characteristic absorption of QUE [21,23], suggesting the successful loading of QUE on the LDHs. In Figure 1b, LDHs show no characteristic absorption peaks in the UV-Vis region, whereas QUE-LDHs show obvious characteristic absorption peaks at 290 and 434 nm. Compared with QUE, the UV-Vis characteristic absorption (at 290 and 434 nm) of QUE-LDHs shows an obvious redshift, which is due to the formation of QUE-Cu coordination compounds [24,25]. Moreover, Cu^2+^ is further confirmed to be involved in the formation of coordination compounds using XPS, and the relevant analysis is supplemented in the Supporting Information.

### 3.2. Microscopic Morphology of QUE-LDHs

**Figure 2:**
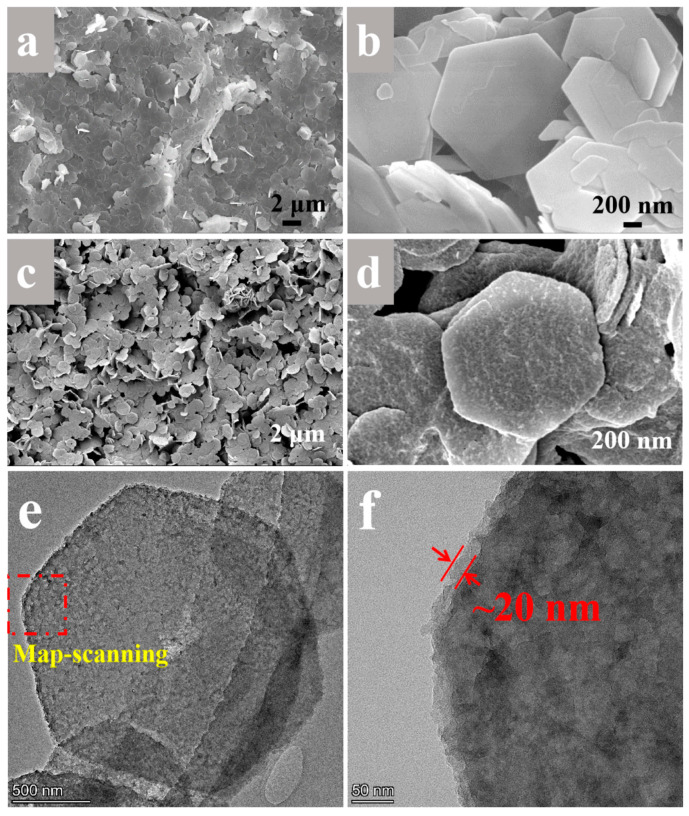
SEM images of (**a**,**b**) LDHs and (**c**,**d**) QUE-LDHs. (**e**,**f**) TEM images of QUE-LDHs.

The microscopic morphology of QUE-LDHs was characterized using TEM and SEM, as shown in Figure 2. In Figure 2a,b, the original LDHs exhibit a typical hexagonal morphology with a smooth surface. After being functionalized with QUE-Cu coordination compounds, LDHs becomes significantly rougher and the edges and corners become more rounded. Furthermore, QUE-LDH nanosheets exhibit uniform dispersion without agglomeration. In order to further confirm the existence of the functional layer, TEM was applied to study the microscopic morphology of QUE-LDHs. In Figure 2e, QUE-LDHs still exhibit a typical hexagonal structure and rough surface. In Figure 2f, a functional layer with a thickness of ~20 nm is formed on the surface of LDHs through adsorption and complexation to form QUE-Cu^2+^ coordination compounds.

### 3.3. Antibacterial Activity of QUE-LDHs

**Figure 3:**
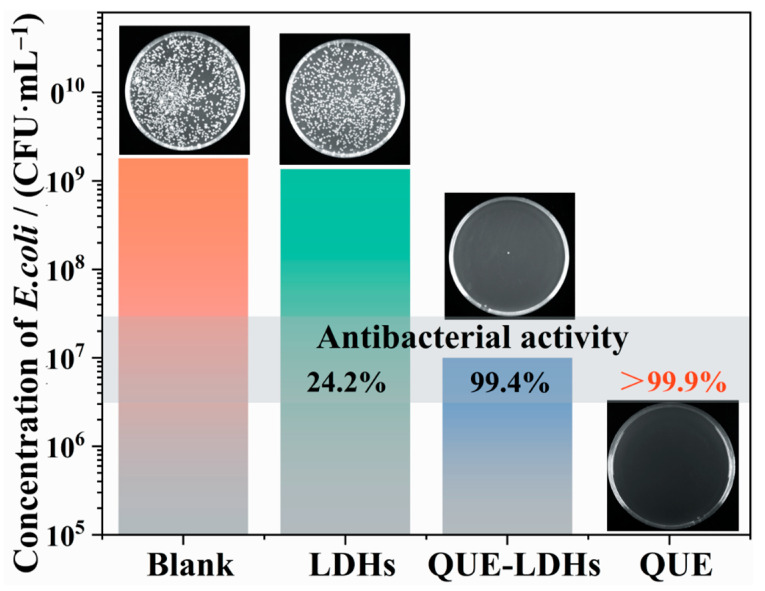
Antibacterial activity against *E. coli* of QUE-LDHs, QUE and LDHs.

QUE, as a kind of plant secondary metabolite, affects the formation of microbial biofilm via its polyphenol structure, hindering the synthesis of enzymes related to metabolism, thereby increasing the permeability of biofilm, which ultimately leads to cell rupturing [21,23]. Figure 3 shows the antibacterial activity against *Escherichia coli* (*E. coli*) of QUE-LDHs, QUE and LDHs. As shown in Figure 3, the antibacterial activity of LDHs is only 24.2%, and there is no obvious antibacterial activity. After the functionalization of QUE-Cu coordination compounds, the antibacterial activity of QUE-LDHs reaches 99.4%. QUE-LDHs exhibit excellent antibacterial activity due to the natural broad-spectrum antibacterial activity of QUE and copper ions [26].

### 3.4. Chemical Structure of QUE-LDHs/CS/PVA Nanocomposite Active Films

**Figure 4:**
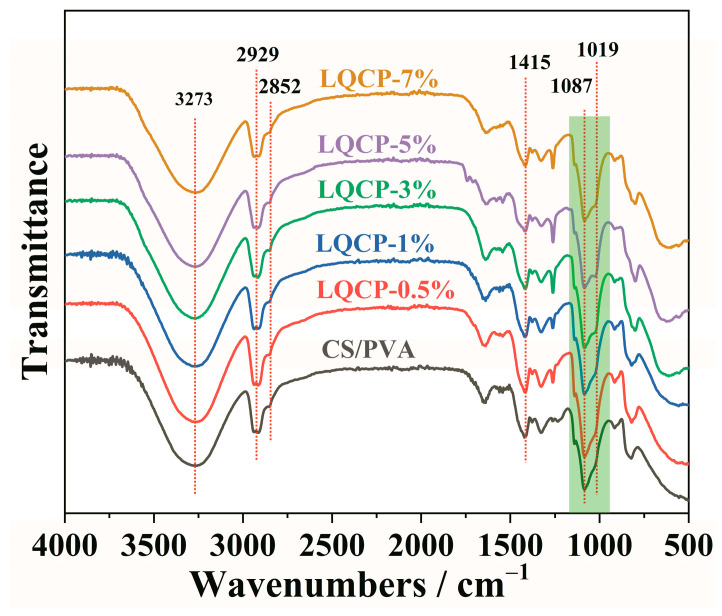
FT-IR spectra of QUE-LDHs/CS/PVA nanocomposite active films.

The FT-IR spectra of QUE-LDHs/CS/PVA nanocomposite active films are illustrated in Figure 4. The absorption peak at 3273 cm^−1^ in the spectrum of the CS/PVA matrix is attributed to the stretching vibration of -OH and -NH groups involved in hydrogen bonds in the CS/PVA matrix [9]. The peaks at 2929, 2852, and 1415 cm^−1^ are due to the stretching and deformation vibrations of C-H bonds [7]. Additionally, the absorption peaks at 1087 and 1019 cm^−1^ correspond to the stretching vibration of C-O bonds [5,13]. After the addition of QUE-LDHs, compared with CS/PVA matrix, there is no significant change in the position and intensity of the characteristic absorption peak for QUE-LDHs/CS/PVA nanocomposite active films. However, subtle changes are observed in the infrared spectral fingerprint range of 1200–950 cm^−1^ (marked in green). In particular, the relative intensity of the absorption peaks at 1087 and 1019 cm^−1^ changes, which reveals that the formation of the hydrogen bond between C-O groups (in the CS/PVA matrix) and phenol hydroxyl groups (in the QUE) causes the change of the infrared peak for C-O groups [27,28]. These changes suggest a strong interaction between QUE-LDHs and CS/PVA matrix, leading to the formation of new hydrogen bonds in the QUE-LDHs/CS/PVA nanocomposite active films.

### 3.5. Thermal Stability of QUE-LDHs/CS/PVA Nanocomposite Active Films

**Figure 5:**
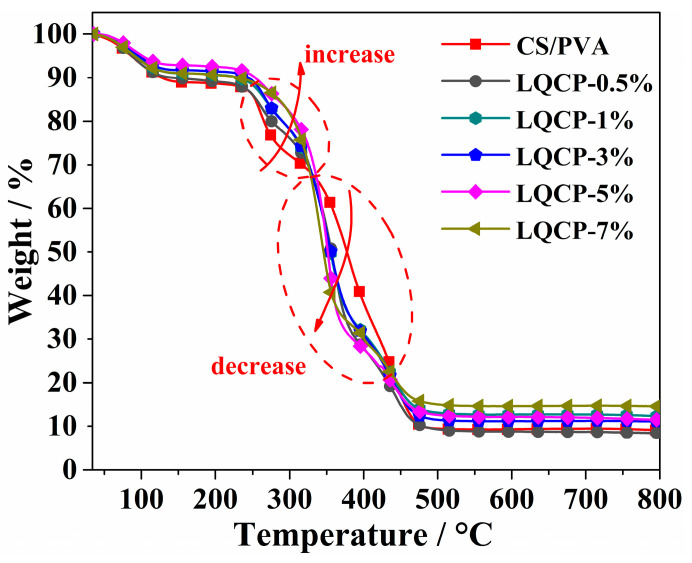
TG curves of QUE-LDHs/CS/PVA nanocomposite active films.

The thermogravimetric curves and thermal stability parameters of the QUE-LDHs/CS/PVA nanocomposite active films are illustrated in Table 2 and Figure 5, respectively. It is observed from Table 2 that, with the addition of QUE-LDHs, the initial decomposition temperature (*T*_−5%_) of QUE-LDHs/CS/PVA nanocomposite active films initially increases and then decreases. This phenomenon is attributed to the enhanced insulation provided by the layered structure of QUE-LDHs [29]. When the addition of QUE-LDHs reaches 5 wt%, *T*_−5%_ reaches a maximum of 101.6 °C. Although *T*_−5%_ starts to decrease with the further addition of QUE-LDHs, it remains higher than that of CS/PVA matrix. In contrast to *T*_−5%_, the temperature at 50% weight loss (*T*_−50%_) consistently decreases with the increase in QUE-LDHs. When the addition of QUE-LDHs is only 0.5 wt%, *T*_−50%_ decreases from 377.3 °C (CS/PVA matrix) to 356.5 °C, showing a decrease of 20.8 °C. As the addition of QUE-LDHs increases from 0.5 wt% to 7 wt%, the decrease in *T*_−50%_ is only 10.8 °C.

Figure 5 indicates that all the films exhibit similar thermal degradation behaviors with two obvious weight loss steps. From room temperature to ~150 °C, QUE-LDHs/CS/PVA nanocomposite active films lose free water and adsorbed water. When the temperature is between ~150 °C and ~230 °C, the weight of QUE-LDHs/CS/PVA nanocomposite active films remains constant. When the temperature is between ~230 °C and 500 °C, the weight loss of QUE-LDHs/CS/PVA nanocomposite active films increases rapidly, indicating the main thermal degradation stage. It is worth noting that the weight loss of QUE-LDHs/CS/PVA nanocomposite active films is always lower than that of CS/PVA matrix before being heated to ~325 °C, which is related to the barrier effect of QUE-LDHs [30]. However, between ~325 °C and ~435 °C, the weight loss of QUE-LDHs/CS/PVA nanocomposite active films surpasses that of CS/PVA matrix, indicating a faster thermal degradation rate, which is due to the co-thermal degradation of QUE and LDHs [21,31]. When the temperature exceeds 435 °C, the weight loss of QUE-LDHs/CS/PVA nanocomposite active films begins to be lower than that of CS/PVA matrix, resulting in a final residual (at 800 °C) being higher than that of CS/PVA matrix. This is because most of the thermal degradation of QUE-LDHs has been completed [32]. Therefore, an appropriate amount (1–5 wt%) of QUE-LDHs can enhance the initial thermal stability of CS/PVA matrix.

### 3.6. Thermal and Crystalline Properties of QUE-LDHs/CS/PVA Nanocomposite Active Films

**Figure 6:**
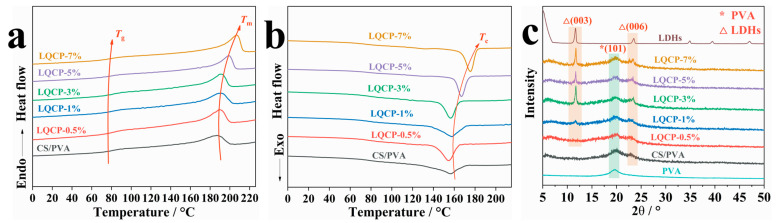
(**a**) DSC second heating curves, (**b**) DSC cooling curves, (**c**) XRD patterns of QUE-LDHs/CS/PVA nanocomposite active films.

The thermal and crystallization properties of QUE-LDHs/CS/PVA nanocomposite active films are characterized by DSC and XRD, as illustrated in Figure 6 and Table 2. In Figure 6a, the glass transition temperature (*T*_g_) and melting temperature (*T*_m_) of QUE-LDHs/CS/PVA nanocomposite active films gradually increase with the addition of QUE-LDHs. This indicates a strong interfacial interaction between QUE-LDHs and CS/PVA matrix, which weakens the mobility of PVA molecules [33,34]. Additionally, combined with a thermal stability analysis of the TG test, the barrier effect of QUE-LDHs prolongs the melting time of CS/PVA matrix. Figure 6b and Table 2 illustrate that the crystallization temperature (*T*_c_) and melting enthalpy (Δ*H*_m_) of all the films show a gradual increase. The addition of QUE-LDHs promotes the rearrangement and stacking of PVA molecules, playing a certain degree of heterogeneous nucleation. However, with the increase in QUE-LDHs, the crystallinity (χ) varies from 19.8% to 21.6%. This indicates that the overall crystallinity remains relatively stable, which may be due to the comprehensive effect of heterogeneous nucleation and interfacial interaction [35,36]. Combined with Figure 6a,b, the advance of crystallization indicates that PVA molecules can form a large number of crystallization nucleation sites on the surface of QUE-LDHs, resulting in an increase in *T*_c._ However, the strong interaction between QUE-LDHs and CS/PVA matrix limits the growth of crystal nuclei, reducing heterogeneous nucleation efficiency [29]. In Figure 6c, characteristic diffraction peaks of (003) and (006) for QUE-LDHs appear in QUE-LDHs/CS/PVA nanocomposite active films around 2θ = 11.7° and 23.5° [37]. The intensity of these peaks increases with the addition of QUE-LDHs, which indicates that the mass fraction of QUE-LDHs increases. Additionally, all the films exhibit a characteristic diffraction peak of (101) at θ = 19.6°, which is attributed to X-ray diffraction of PVA [38]. However, the (101) crystal plane of PVA does not change significantly with the addition of QUE-LDHs, which is similar to the crystallinity change in the DCS analysis.

### 3.7. Mechanical Properties of QUE-LDHs/CS/PVA Nanocomposite Active Films

**Figure 7:**
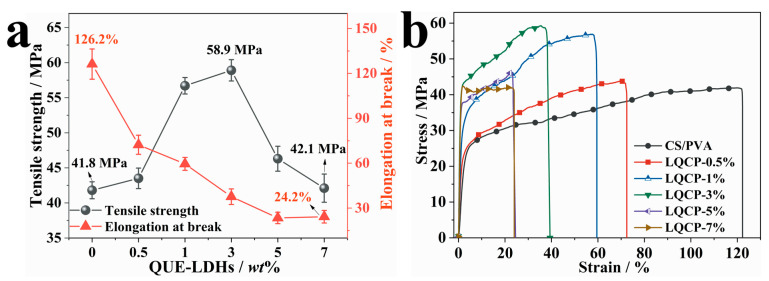
(**a**) Mechanical properties and (**b**) stress–strain curvesof QUE-LDHs/CS/PVA nanocomposite active films.

Figure 7 illustrates the mechanical properties and stress–strain curves of QUE-LDHs/CS/PVA nanocomposite active films. In Figure 7a, the tensile strength and elongation at break of CS/PVA matrix are 41.8 MPa and 126.2%, respectively. When the addition of QUE-LDHs reaches 3 wt%, the tensile strength achieves the maximum value of 58.9 MPa, representing a significant 40.9% increase compared with CS/PVA matrix (41.8 MPa). Although the tensile strength begins to decrease continuously with the addition of QUE-LDHs, the tensile strength of LQCP-7% (42.1 MPa) does not change significantly compared with CS/PVA matrix. In contrast to the changes in the tensile strength, the elongation at break gradually decreases with the increase in QUE-LDHs. When the molecular chain slides under stress, the chain mobility of CS/PVA matrix is decreased due to the limitation of strong interface interactions caused by hydrogen bondings, ultimately leading to premature fracture of the film [5,39]. Meanwhile, according to DSC test results, the existence of QUE-LDHs increases χ of CS/PVA matrix, further decreases the mobility of molecular chains.

As shown in Figure 7b, the stress–strain curve of CS/PVA matrix are similar to those of pure PVA, with no obvious yield point and strain softening stage [40]. With the addition of QUE-LDHs (no more than 3 wt%), the yield strength and breaking strength of QUE-LDHs/CS/PVA nanocomposite active films show an increasing trend, which indicates that QUE-LDHs play a significant enhancement effect. When the addition of QUE-LDHs reaches 5 wt%, the strength of QUE-LDHs/CS/PVA nanocomposite active films begins to decrease significantly, which is related to the decrease in enhancement efficiency and agglomeration of QUE-LDHs. The strain decreases with the increase in QUE-LDHs, which reveals that the existence of QUE-LDHs weakens the interactions between CS and PVA.

**Figure 8:**
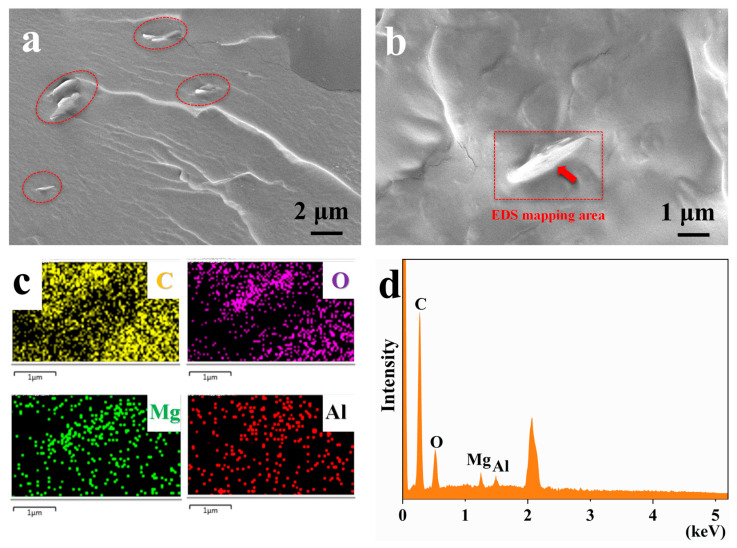
(**a**,**b**) SEM images for fracture surface, (**c**) EDS map-scanning, (**d**) EDS spectrum of LQCP-3%.

In order to further analyze the reasons for the changes of the mechanical properties, the microscopic morphology of the fracture surface was investigated, as shown in Figure 8. As shown in Figure 8a,b, QUE-LDHs is uniformly dispersed at nanometer size in CS/PVA matrix without obvious agglomeration, and there are no visible defects or gaps between QUE-LDHs and CS/PVA matrix. This reveals that QUE-LDHs and CS/PVA matrix have good interface compatibility, which helps to enhance the strength of CS/PVA matrix. The EDS element analysis of the marked region in Figure 8b, as presented in Figure 8c,d, further confirms the presence of QUE-LDHs.

### 3.8. Optical Properties of QUE-LDHs/CS/PVA Nanocomposite Active Films

**Figure 9:**
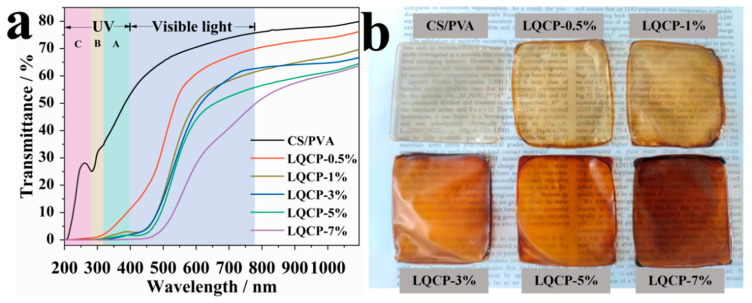
(**a**) UV-Vis light transmittance and (**b**) digital photographs of QUE-LDHs/CS/PVA nanocomposite active films.

Figure 9 displays the UV-Vis light transmittance and digital photographs of QUE-LDHs/CS/PVA nanocomposite active films. Good UV barrier properties can effectively delay the deterioration of food under UV radiation, ensuring the quality of food during storage and transportation, and prolonging the shelf life of food [41,42]. As shown in Figure 9a, the UV transmittance of CS/PVA matrix at 280 nm and 400 nm is only 25.3% and 53.2%, demonstrating certain UV barrier properties. It indicates that CS/PVA matrix already exhibits significant absorption in the UV light region (190–400 nm), which is caused by the catechol structure and unsaturated bonds in CS [22]. With the addition of QUE-LDHs, the UV absorption of QUE-LDHs/CS/PVA nanocomposite active films significantly increases, which is attributed to the large amount of active catechol groups in QUE-LDHs. When the addition of QUE-LDHs is only 1 wt%, the UV transmittance of LQCP-1% at 280 nm and 400 nm reduces to 0.1% and 2.9%, which is a 99.6% and 94.5% lower than that of CS/PVA matrix, indicating excellent UV barrier properties. In our previous study [35], when the same amount (1 wt%) of curcumin-functionalized LDHs was added to CS/PVA matrix, the UV transmittance at 280 nm and 400 nm was reduced by only 70.4% and 35.3%. This indicates that QUE-LDHs contains a large number of active catechol groups, which show better UV barrier properties, while curcumin-functionalized LDHs only contain a large number of active phenolic hydroxyl groups. With further addition of QUE-LDHs (3–7 wt%), the UV barrier properties reach 100% at 280 nm. In the visible light region (400–780 nm), the light transmittance at 600 nm of CS/PVA film is 70.5%. With the addition of QUE-LDHs, the light transmittance decreases gradually. When the addition of QUE-LDHs reaches 7 wt%, the reduction in light transmittance at 600 nm reaches 60.7% compared with CS/PVA matrix. When the same amount (7 wt%) of curcumin-functionalized LDHs was added to CS/PVA matrix, the light transmittance at 600 nm is reduced by only 29.8% [35]. This suggests that QUE-LDHs have a greater effect on visible light transmittance. However, the absorption of UV light is much greater than that of visible light. Meanwhile, the addition of QUE-LDHs can significantly enhance the high-energy blue light (400–500 nm) barrier properties of CS/PVA matrix. The barrier of high-energy blue light is also crucial for food preservation, as it helps delay the photooxidation of organic compounds and the degradation of vitamins and other pigments [43].

Table 3 presents the optical properties of QUE-LDHs/CS/PVA nanocomposite active films. As shown in Table 3, the opacity of QUE-LDHs/CS/PVA nanocomposite active films gradually decreases with the addition of QUE-LDHs. When the addition of QUE-LDHs does not exceed 5 wt%, the opacity is less than five, indicating that QUE-LDHs/CS/PVA nanocomposite active films exhibit transparency. Providing a clear view of food and packaging conditions while providing good UV and high-energy blue light barrier properties are prerequisite for excellent food packaging film materials [13]. As shown in Figure 9b, the color of QUE-LDHs/CS/PVA nanocomposite active films shows a trend from colorless to brown. Despite the deepening color, the text behind QUE-LDHs/CS/PVA nanocomposite active films (the addition of QUE-LDHs does not exceed 5 wt%) remains clearly visible, demonstrating a certain degree of transparency, which is consistent with the opacity results. The colorimeter was used to further accurately analyze the color change of QUE-LDHs/CS/PVA nanocomposite active films, as shown in Table 3. It is evident that with the increase in QUE-LDHs, the L* (brightness) significantly decreases, which is correlating with the decrease in visible light transmittance. Influenced by the color of QUE-LDHs, the values of a* and b* continue to increase, indicating that QUE-LDHs/CS/PVA nanocomposite active films exhibit a trend of reddening and yellowing. Due to the growing color difference between the films and standard whiteboard, ΔE* increases from 21.6 to 84.8. In our previous study [35], the addition of curcumin-functionalized LDHs resulted in an increase in ΔE* from 21.6% to 59.1%. The results indicate that the addition of QUE-LDHs has a significant effect on the color of CS/PVA matrix.

### 3.9. Antibacterial Activity of QUE-LDHs/CS/PVA Nanocomposite Active Films

**Figure 10:**
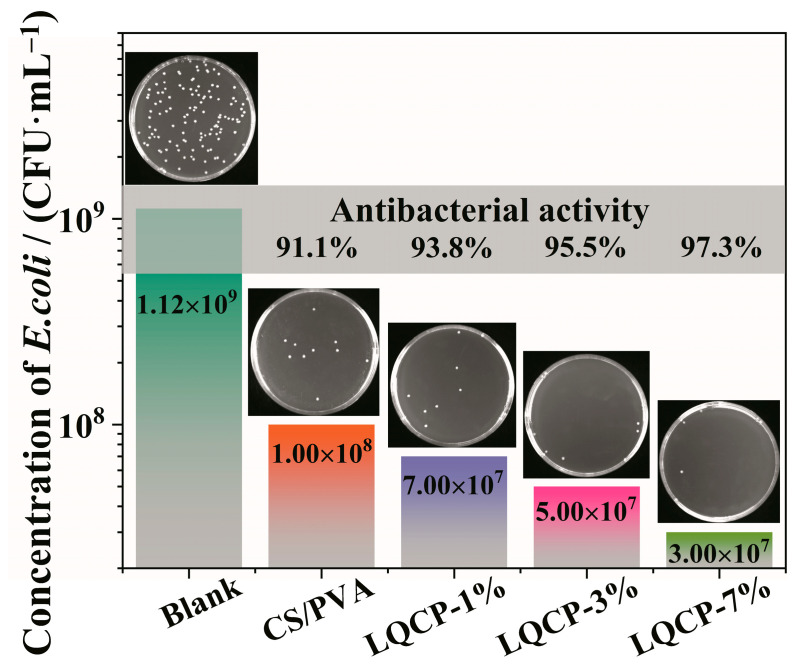
Antibacterial activity against *E. coli* of QUE-LDHs/CS/PVA nanocomposite active films.

Figure 10 shows the antibacterial activity against *E. coli* of QUE-LDHs/CS/PVA nanocomposite active films. The antibacterial activity of CS/PVA matrix reaches 91.1%, showing good antibacterial ability, which is due to the excellent broad-spectrum antibacterial activity of CS [6]. According to literature reports, the antibacterial mechanism for CS is due to the strong interaction between positively charged amino groups of CS and negatively charged membranes of bacterial, resulting in increased membrane permeability and the leakage of intracellular components, and ultimately leading to bacterial death [6,44]. The antibacterial activity of QUE-LDHs/CS/PVA nanocomposite active films exhibits a gradual increase trend upon the increase in QUE-LDHs. When the addition of QUE-LDHs reaches 7 wt%, the antibacterial activity of LQCP-7% reaches the maximum of 97.3%, showing excellent antibacterial ability. These results indicate that the presence of active QUE-LDHs can enhance the antibacterial ability of CS/PVA matrix.

### 3.10. Antioxidant Activity of QUE-LDHs/CS/PVA Nanocomposite Active Films

**Figure 11:**
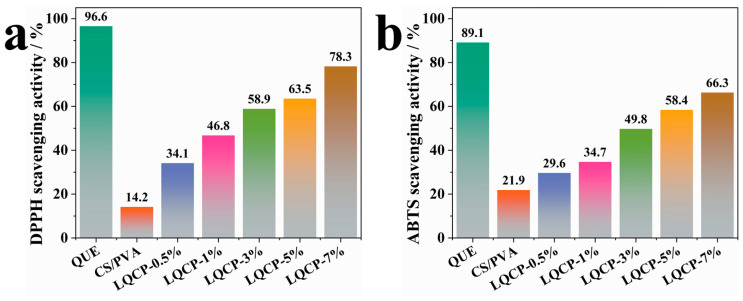
Antioxidant activity of QUE-LDHs/CS/PVA nanocomposite active films ((**a**), DPPH mehod. (**b**), ABTS method).

The antioxidant ability of food packaging materials can effectively prevent the oxidation of nutrients in food and reduce the loss of food quality [45]. DPPH and ABTS radical scavenging activities were applied to evaluate the antioxidant ability of QUE-LDHs/CS/PVA nanocomposite active films, as shown in Figure 11. In Figure 11a, DPPH radical scavenging activity of QUE reaches 96.6%, showing excellent antioxidant activity, which is due to the large number of active groups in QUE effectively trapping radicals and preventing the oxidation reaction of nutrients [21,23]. CS/PVA matrix does not have sufficient active groups, therefore it cannot effectively scavenge DPPH radicals, and its antioxidant activity is only 14.2%. With the addition of QUE-LDHs, its antioxidant activity gradually increases. When the amount of QUE-LDHs reaches 7 wt%, the antioxidant activity reaches the maximum value of 78.3%. ABTS radical scavenging activity experiment also shows the same trend. However, it can be seen from radical scavenging experiments that the scavenging activity of DPPH radical is significantly higher than that of ABST radical under the same amount of QUE-LDHs. This is because the solvent used in DPPH radical scavenging activity experiment is alcohol solution, while ABTS radical scavenging activity experiment is aqueous solution. Additionally, QUE is almost insoluble in water, but soluble in ethanol.

## 4. Conclusions

In this study, QUE-LDHs realizes a combination of active functions and enhancement effect through the deposition and complexation of QUE and copper ions on the LDHs’ surface. CS/PVA matrix, reinforced with active QUE-LDHs, was prepared by a solution casting method. The results show that QUE-Cu^2+^ coordination compounds successfully to functionalize the LDHs nanosheets with a functional layer thickness of ~20 nm. Infrared and thermal analysis results revealed that there was a strong interface interaction between QUE-LDHs and CS/PVA matrix, resulting in the limited movement of PVA molecular chains and the increase in glass transition temperature and melting temperature. The thermal stability analysis revealed that the addition of QUE-LDHs can increase the initial decomposition temperature to a certain extent, but it still increased the thermal degradation rate of CS/PVA matrix in the later stage. After the addition of QUE-LDHs, QUE-LDHs/CS/PVA nanocomposite active films showed an excellent UV barrier, antibacterial, antioxidant properties and tensile strength, and still had certain transparency in the range of visible light. As the QUE-LDH content was 3 wt%, the active films exhibited the maximum tensile strength of 58.9 MPa, representing a significant increase in 40.9% compared with CS/PVA matrix. Notably, the UV barrier (280 nm) and antibacterial (*E. coli*) and antioxidant activities (DPPH method) of the active films achieved 100.0%, 95.5% and 58.9%, respectively. Therefore, LDHs@QUE-Cu/CS/PVA nanocomposite active films show excellent active packaging functions and have good potential in extending the shelf life of food.

## Figures and Tables

**Table 1:** The composition and abbreviation of QUE-LDHs/CS/PVA nanocomposite active films.

Samples	PVA/g	CS/g	QUE-LDHs/g	QUE-LDHs/wt%
CS/PVA	0.63	0.07	0	0
LQCP-0.5%	0.63	0.07	0.0035	0.5%
LQCP-1%	0.63	0.07	0.0071	1%
LQCP-3%	0.63	0.07	0.0216	3%
LQCP-5%	0.63	0.07	0.0368	5%
LQCP-7%	0.63	0.07	0.0526	7%

**Table 2:** Thermal analysis results of QUE-LDHs/CS/PVA nanocomposite active films.

Sample	*T*_−5%_/°C	*T*_−50%_/°C	*T*_g_/°C	*T*_m_/°C	*T*_c_/°C	Δ*H*_m_/(J/g)	χ/%
CS/PVA	86.6	377.3	78.1	186.7	156.6	23.56	16.1%
LQCP-0.5%	87.3	356.5	81.5	189.5	154.5	29.40	20.1%
LQCP-1%	92.9	355.2	81.8	191.4	157.6	29.69	20.4%
LQCP-3%	95.8	355.6	81.3	190.4	156.6	28.21	19.8%
LQCP-5%	101.6	351.4	79.7	199.1	166.9	30.11	21.6%
LQCP-7%	90.6	345.7	79.8	204.5	173.5	27.50	20.2%

**Table 3:** Optical properties of QUE-LDHs/CS/PVA nanocomposite active films.

Sample	T_280_/% ^1^	T_400_/% ^1^	T_600_/% ^1^	Thickness/mm	Abs_600_^2^	Opacity	L*	a*	b*	Δ*E*
CS/PVA	25.3	53.2	70.5	0.091	0.152	1.670	81.9 ± 0.1	1.9 ± 0.2	15.7 ± 0.2	21.6 ± 0.4
LQCP-0.5%	0.6	11.3	60.8	0.073	0.216	2.959	76.8 ± 0.2	6.0 ± 0.1	40.4 ± 0.4	45.4 ± 0.3
LQCP-1%	0.1	2.9	50.1	0.071	0.300	4.225	73.4 ± 0.1	6.9 ± 0.3	42.2 ± 0.2	46.9 ± 0.3
LQCP-3%	0	2.0	47.3	0.075	0.325	4.333	56.5 ± 0.2	21.0 ± 0.4	48.5 ± 0.4	66.4 ± 0.5
LQCP-5%	0	1.7	44.0	0.072	0.357	4.958	55.8 ± 0.3	26.0 ± 0.5	52.2 ± 0.6	71.3 ± 0.5
LQCP-7%	0	0.20	27.7	0.075	0.558	7.440	41.0 ± 0.5	24.6 ± 0.3	59.3 ± 0.2	84.8 ± 0.4

^1^ Light transmittance at 280, 400, 600 nm. ^2^ Absorbance at 600 nm.

## Data Availability

Data are contained within the article and Supplementary Materials.

## Supplementary Materials

The following supporting information can be downloaded at: https://www.mdpi.com/article/10.3390/polym16060727/s1. Figure S1: XPS spectra of LDHs and QUE-LDHs. References [46,47,48] are cited in the supplementary materials.
