# Supplementary material for: Development of Sustainable and Active Food Packaging Materials Composed by Chitosan, Polyvinyl Alcohol and Quercetin Functionalized Layered Clay
Source: Polymers (Basel). 2024 Mar 7;16(6):727. doi: 10.3390/polym16060727 (PMC10974495; doi:10.3390/polym16060727)
Supplement: Supplementary file 1 [file polymers-16-00727-s001.zip › polymers-2892720-supplementary.pdf]

Development of sustainable and active food packaging materials composed by chitosan, polyvinyl alcohol, quercetin functionalized layered clay

Chengyu Wang [1], Long Mao [1,2*], Bowen Zheng[2], Yujie, Liu[2], Jin Yao [1], Heping Zhu [1*]

[1] *Key Laboratory of Advanced Packaging Materials and Technology of Hunan Province, Hunan University of Technology, Zhuzhou 412007, China*

[2] *Fujian Provincial Key Laboratory of Functional Materials and Applications, Xiamen University of Technology, Xiamen 361024, China*

* Correspondence: maolong@xmut.edu.cn (L. Mao) and zhuheping@hut.edu.cn (H.Z.)

Content

Characterization

Fourier transform infrared (FT-IR) spectra were conducted on the wavenumber of 4000–400 cm^{-1} using a FT-IR spectrophotometer (ALPHA, Bruker) under attenuated total reflection mode (ATR). The samples were placed directly on the sample holder and scanned at the resolution of 4 cm^{-1}.

Ultraviolet-visible (UV-vis) spectra were recorded on an UV–vis spectrophotometer (SPECORD 210 PLUS, Analytikjena) in the wavelength range of 190-800 nm. Opacity of the films was calculated as follows, opacity = Abs_{600}/t, where Abs_{600} and t are the absorbance value at 600 nm and the thickness (mm) of each film, respectively [13].

X-ray photoelectron spectroscopy (XPS) spectra were carried out on a spectrophotometer (AXIS SUPRA$^+$, Kratos) using an Al Kα X-ray source (30 eV pass energy and 400 μm spot size).

The microscopic morphology of surface and fracture surface for the samples was observed using field-emission scanning electron microscope (FE-SEM, sigma500, Zeiss) after sputtering gold onto the samples. Field-emission transmission electron microscope (FE-TEM, Talos, FEI) was used to observe the morphology of QUE-LDHs at high magnification. Further elemental analysis was carried out with an energy dispersive X-ray spectrometer (EDS, X-Max^n, Oxford) equipped with FE-SEM.

The antibacterial activity against *Escherichia coli* (*E. coli*) was evaluated by plate count method with antibacterial rate as the index. Specific experimental methods referred to our previous study [22]. The antibacterial rate was calculated as follows, the antibacterial

rate = [($N_{control}$ − N_{sample})/$N_{control}$]×100%, where N_{sample} is value of colony forming units (CFU/mL) for each sample, and $N_{control}$ is value of colony forming units for blank sample.

Thermal stability of the samples was carried out on a thermal gravimetric analyzer (TGA) (TG209F3, Netzsch). The heating rate was 10 °C/min in the temperature range from 35 °C to 1000 °C under N_2 atmosphere. $T_{-5\%}$ and $T_{-50\%}$, defined as the temperature at 5% mass loss and 50% mass loss respectively, were measured.

Differential scanning calorimeter (DSC) (DSC214, Netzsch) was applied to analyze the thermal behavior of the samples under N_2 atmosphere. The heating and cooling procedures referred to our published paper [20]. The crystallinity (χ) of PVA is calculated by the following formula, $\chi=[\Delta H_m/(\Delta H_0\times\varphi)]\times100\%$, where ΔH_m was the experimental melting enthalpy of the samples, ΔH_0 was the melting enthalpy of 100% crystalline PVA (163 J/g) [33], and φ was the mass fraction of PVA in QUE-LDHs/CS/PVA nanocomposite active films.

X-ray diffraction (XRD) patterns were recorded on a diffractometer (SmartLab 3KW, Rigaku), using CuKα radiation at a scanning rate of 5 °/min (from 5 ° to 50 °).

The color of the films was evaluated by a chromameter (TS7020, Threenh) under CIE standard D65/10°. A white color plate (L^*=96.9, a^*=−0.2, b^*=0.3) was used as a standard for analysis. The color of samples was expressed as L^*(lightness), ±a^* (redness/greenness), and ±b^*(yellowness/blueness) values. Analysis of color difference (ΔE^*) was evaluated as follows, $\Delta E^*=(\Delta L^{*2}+\Delta a^{*2}+\Delta b^{*2})^{1/2}$, where ΔL^*, Δa^* and Δb^* are the differences of color parameter between the films and the standard white plate [8]. Each sample was measured at least three times. The mean value and standard deviation for each film were calculated according to experimental results.

The mechanical properties of the films were tested by micro-controlled electronic universal testing machine (ETM502B-Ex, Wance) at a tensile rate of 10 mm/min. Measurements were repeated five times for each film. The mean value and standard deviation for each film were calculated according to experimental results.

The antioxidant assessment was evaluated by DPPH radical scavenging method with slight adjustment [8]. Briefly, the film was cut into a size of 2 cm×2 cm (or 3 mg of QUE), put into each beaker filled with 20 mL of deionized water, and stirred at room temperature for 24 h. About 1 mL of the above solution was taken out to be mixed with 4 mL of methanol solution of DPPH (75 μmol/L) under stationary condition for 1 h. The absorbance of the above mixed solution was measured at 520 nm by using a UV-Vis spectrophotometer. DPPH scavenging activity (%) was calculated according to the

following formula: DPPH scavenging activity (%) = (1 － $Abs_{sample}/Abs_{control}$)×100%, where $Abs_{control}$ is the absorbance value of deionized water mixed with methanol solution of DPPH, Abs_{sample} is the absorbance value of sample solution mixed with methanol solution of DPPH.

The antioxidant assessment was also evaluated by ABTS radical scavenging method with slight adjustment [46]. Briefly, the film was cut into a size of 2 cm×2 cm (or 3 mg of QUE), put into each beaker filled with 20 mL of deionized water, and stirred at room temperature for 24 h. ABTS solution was generated by dissolving 0.3841 g of ABST and 0.0662 g of potassium persulfate successively in 100 mL of deionized water. The final concentration ABTS and potassium persulfate were 7.00 mmol/L and 2.45 mmol/L, respectively. ABTS solution was magnetically stirred at room temperature for 16h in the dark. Subsequently, ABTS solution was diluted in phosphate buffer solution to an absorbance of 0.70 ± 0.02 at λ = 734 nm. About 0.3 mL of sample solution was taken out to be mixed with 4 mL of ABTS solution under stationary condition for 6 min. The absorbance of the above mixed solution was measured at 734 nm by using a UV-Vis spectrophotometer. ABTS scavenging activity (%) was calculated according to the following formula: ABTS scavenging activity (%) = (1 － $Abs_{sample}/Abs_{control}$)×100%, where $Abs_{control}$ is the absorbance value of deionized water mixed with ABTS solution, Abs_{sample} is the absorbance value of sample solution mixed with ABTS solution.

Results and Discussion

As shown in Figure 1S, the peak which is attributed to Cu $2p_{3/2}$ is found in the spectrum of QUE-Cu and the mass fraction of Cu element is 1.8% [47]. From the high-resolution XPS spectrum of Cu $2p_{3/2}$ (Figure 1S inset), two peaks appear at 935.6 eV and 933.8 eV. According to the intensity and area of energy spectrum peak, it can be inferred that the main energy spectrum peak of Cu$2p_{3/2}$ appears at 933.8 eV, which is obviously lower than Cu ions in the free state (935.6 eV) [19]. The reduced binding energy of Cu is caused by the acceptance of electrons by Cu ions during the formation of QUE-Cu^{2+} complex [47,48]. Moreover, XPS elemental analysis results reveal that the mass ratio of C/O (0.83) in the QUE-LDHs is much higher than that (~0.36) [19] in the LDHs.

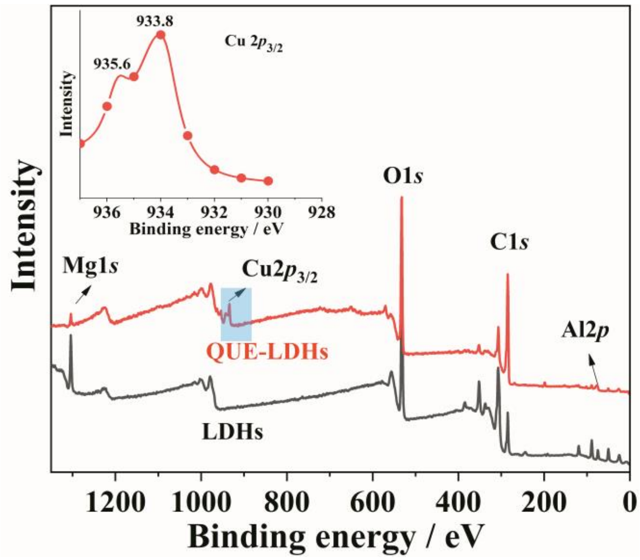

Figure S1. XPS spectra of LDHs and QUE-LDHs

References

References in the supporting information are available in the manuscript.
